# THBS2 is a Potential Prognostic Biomarker in Colorectal Cancer

**DOI:** 10.1038/srep33366

**Published:** 2016-09-16

**Authors:** Xue Wang, Lei Zhang, Hui Li, WenJie Sun, Honghe Zhang, Maode Lai

**Affiliations:** 1 Department of Pharmacology, China Pharmaceutical University, Nanjing, 210009, China; 2 Department of Pathology, School of Medicine, Zhejiang University, Hangzhou 310058, China; 3 Key Laboratory of Disease Proteomics of Zhejiang Province, Hangzhou 310058, China

## Abstract

Colorectal cancer is one of the most common leading causes of death worldwide. Prognostic at an early stage is a useful way that decrease and avoid mortality. Although remarkable progress has been made to investigate the underlying mechanism, the understanding of the complicated carcinogenesis process was enormously hindered by large-scale tumor heterogeneity. Here we proposed that the prognosis-related gene THBS2, responsible for cooperativity disorientation, probably contain untapped prognostic resource of colorectal cancer. We originally established Spearman correlation transition, Kaplan–Meier survival analysis and meta-analysis that combine public dataset and clinical samples to quantify the prognostic value of THBS2. THBS2 could be considered as a novel prognostic marker in colorectal cancer.

Colorectal cancer (CRC) is the third most common occurring noncutaneous carcinoma and the third leading cause of cancer-related death worldwide^1^. The patients suffering from CRC can be cured if detected at an early stage. Unfortunately, majority patients could not be accurately diagnosed until advanced stages accompanied with malignant proliferation, extensive invasion, lymph node and distant metastasis^2^. Despite advances have been made by surgical techniques and chemotherapeutic options since the last decades, the survival rate of colorectal cancer seems not to be substantially improved, and approximately 20% of patients die of recurrence and metastasis events accounting for the poor prognostic outcomes^3^. There are not marked variability in outcome exists that can predict the patients who have potential higher risk in recurrence, metastasis, resistance to chemotherapy and decreased survival by using conventional histopathologic staging. Thus, identification of novel prognostic biomarkers in colorectal cancer is vital to develop effective targeted treatment for clinical benefits.

The Gene Expression Omnibus database (GEO)^4^ and The Cancer Genome Atlas (TCGA) are international public repositories that contained a large number of genomic datasets with clinical information. Several genes that have diagnostic value in colorectal cancer have been found by using these datasets^5^,^6^,^7^. While tumor stage, tumor grade, patient age at diagnosis, performance status, race or areas, ethnicity as well as the diet propensity are important factors that may lead a contradistinction phenotype. Therefore, a systematic integration of gene expression data from multiple sources meta-analyses, increase statistical power for detecting differentially expressed datasets while allowing for an assessment of heterogeneity, and may lead to more robust, reproducible and accurate predictions^8^.

Thrombospondins (THBS2) is a multifunction alglycoprotein released from various types of cell, including stromal fibroblasts, endothelial cells and immune cells^9^. THBS2 exerts its diverse biological effects such as angiogenesis, cell motility, apoptosis, cytoskeletal organization by binding with extracellular matrix (ECM) proteins and cell surface receptors^10^,^11^,^12^. THBS2 is also known to regulate the activation of transforming growth factor-β1 (TGF-β1)^13^. Notably, THBS2 is special in this family for their type I repeats and mainly shown as complex function in cancer research^14^,^15^,^16^. Our previous study used biopsy screening and laser capture techniques significant slightly separated rectal cancer anterior single germ of tumor budding, tumor cells and normal mucous cells in fresh colorectal cancer surgical specimens^17^. The results showed that the expression of THBS2 in tumor budding was significantly increased that may closely related with tumor metastasis and prognosis. To verify our aforementioned hypothesis, we examined the THBS2 expression levels in human colorectal cancer tissues and corresponding normal tissues in public datasets and clinical samples. And a comprehensive evaluation by meta-analysis that explored the possible correlation between THBS2 with clinical prognosis in colorectal cancer will be more accuracy.

## Results

### CRC datasets preparation

GSE17536 and corresponding clinical data were downloaded from the publicly available GEO datasets. This dataset include 177 patients, 96 male and 81 female, in total 55 patients were dead during the follow-up time and 122 patients survived. With the filtering condition display recurrence in 145 patients, 36 patients relapse and 109 were non recurrence. 59 recurrence-related differentially expressed genes have been extracted between the recurrence and non-recurrence groups, including 59 up-regulated genes and 3 down-regulated genes (Supplementary Table S1).

### Hierarchical cluster and Neighborhood Scoring

**Figure 1:**
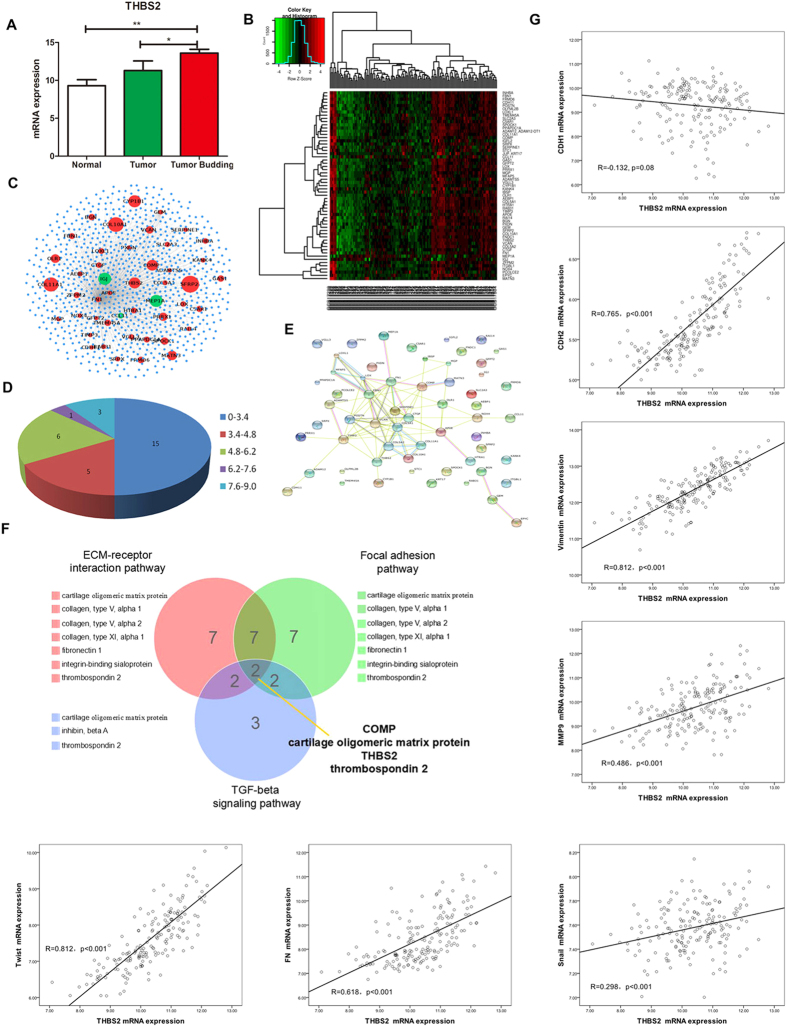
(**A**) The expression of THBS in normal, tumor and tumor budding in CRC. (**B**) Hierarchical clustering of colorectal cancer samples. A green-red heat map was used to represent up- and down-regulated genes visualize the clustering results. (**C**) Protein-protein interaction (PPI) networks of differentially expressed genes (DEGs). (**D**) DEGs degree distribution. (**E**) The interaction of protein by string. (**F**) Pathway enrichment. (**G**) The correlation between THBS2 with EMT marker.

To identify and validate the existence of different genes in recurrence and non-recurrence groups, hierarchical clustering was performed using samples of cancer based on the genes that were identified as differentially expressed in these two subtypes. In order to analyze these 59 genes in combination with biological significance and statistical significance, we mapped these 59 genes to human protein interaction network to establish the interaction among them (Fig. 1B). In the construction of the network, the degree of variation that included distribution and change fold were used to reflect the importance of genes (Fig. 1C–E, Supplementary Table S2). The above result showed that several genes MEP1A, MFAP5, THBS2, VCAN, FBN1 and COMP owned the highest score with important degree node distribution which had interaction with many genes.

### Pathway analysis

DAVID was applied to analysis recurrence associated genes and enrichment on gene functions and pathways. In this study, recurrence associated genes from DEGs were mined by Disease Module to analyze the enrichment genes in KEGG pathway (Fig. 1F, Supplementary Table S3). The genes were mainly enriched in extra-cellular matrix (ECM)-receptor interaction (n = 7, p = 2.70E-07), focal adhesion (n = 7, p = 4.40E-05) and TGF-beta signaling pathway (n = 3, p = 3.70E-02). Extra-cellular matrix (ECM)-receptor interaction pathway include seven genes: COMP, COL5A1, COL5A2, COL11A1, FN1, IBSP and THBS2. Focal adhesion pathway include seven genes: COMP, COL5A1, COL5A2, COL11A1, FN1, IBSP and THBS2. TGF-beta signaling pathway include three genes: COMP, INHBA and THBS2. There exist two genes THBS2 and COMP showed important effect on three pathways which indicated these genes may have important function.

### Hypergeometric Distribution

The hypergeometric distribution test was used to analysis correlation between genes with disease. PubMed comprises more than 25 million citations for biomedical literature from medicine, life science journals and online books that correlated with colorectal cancer or THBS2 is 182043 or 81, respectively. The number of review that correlated with both colorectal cancer and THBS2 is 4. Comp owned 1037 reviews, but only 2 reviews had been reported with colorectal cancer (Supplementary Table S4). The hypergeometric distribution analysis showed that THBS2 have a higher correlation with colorectal cancer when compared with COMP (p = 0.0029). These results indicated that THBS2 maybe owned significant correlation with colorectal cancer.

### Pearson correlation analysis

To better understand the biology of the tumors with high levels of THBS2, we identified gene transcripts that most closely correlate with expression of THBS2 in the GSE17536 dataset by Pearson test^18^. The majority of the THBS2-correlated genes were matricellular-extracellular matrix and focal adhesion genes (Supplementary Table S5). The average of top 20 genes excluded THBS2 showed a high risk in overall survival (p < 0.001, 2.746(1.593–4.734)) which also could be a useful biomarker in CRC. Interestingly, combination the average of top 20 genes and THBS2 showed a higher risk in overall survival (p < 0.001, 2.890(1.676–4.981)) than the average of top 20 gene without THBS2. The results indicated that THBS2 increased HR value, which might be considered as a prognostic biomarker for CRC. Moreover, THBS2 was higher expression in tumor budding, which was considered as the cells with EMT phenotype. There was a significant correlation between THBS2 expression with CDH1, CDH2 and other EMT markers (Fig. 1A,G).

### Kaplan–Meier survival analysis confirmed the prognostic value of THBS2 in GEO and TCGA datasets

**Figure 2:**
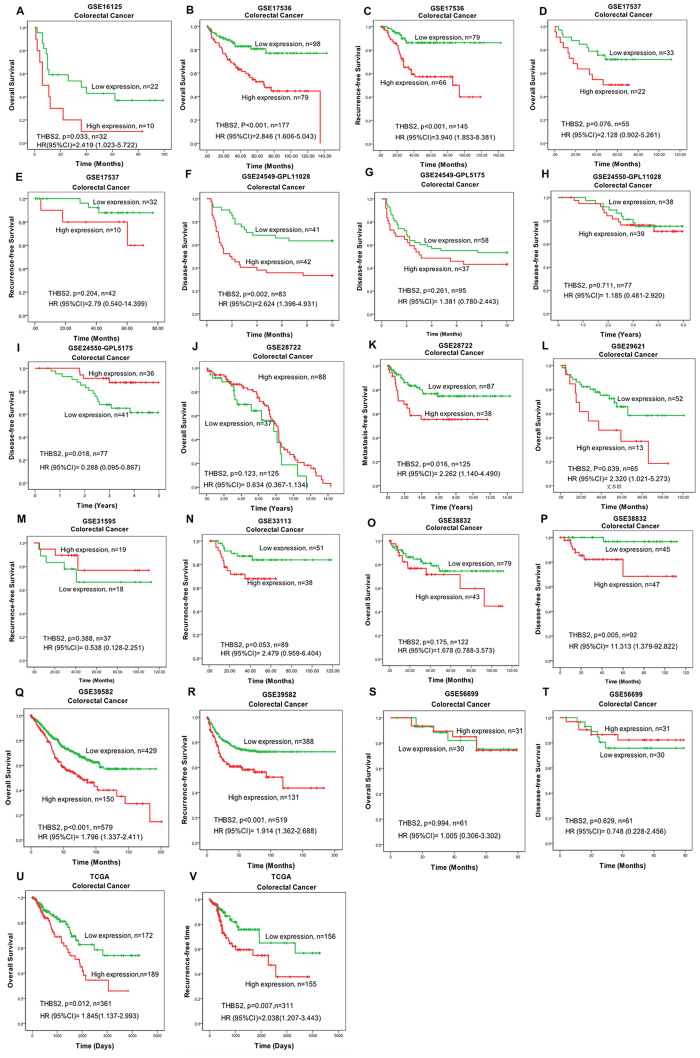
The correlation between THBS2 expression and prognosis overall survival/disease free survival were analyzed by Kaplan-Meier survival curve in GEO datasets. TheTHBS2 level was evaluation by characteristic (ROC) curve. (**A**) GSE16125-OS; (**B**) GSE17536-OS; (**C**) GSE17536-RFS; (**D**) GSE17537-OS; (**E**) GSE17537-RFS; (**F**) GSE24549-GPL11028-DFS; (**G**) GSE24549-GPL5175-DFS; (**H**) GSE24550-GPL5175-DFS; (I) GSE24550-GPL11028-DFS; (**J**) GSE28722-OS; (**K**) GSE28722-MFS; (**L**)GSE29621-OS; (**M**) GSE31595-RFS; (**N**) GSE33113-RFS; (**O**) GSE38832-OS; (**P**) GSE38832-DFS; (**Q**) GSE39582-OS; (**R**) GSE39582-RFS; (**S**) GSE56699-OS; (**T**) GSE56699-DFS; (**U**) TCGA-OS; (**V**) TCGA-RFS.

**Figure 3:**
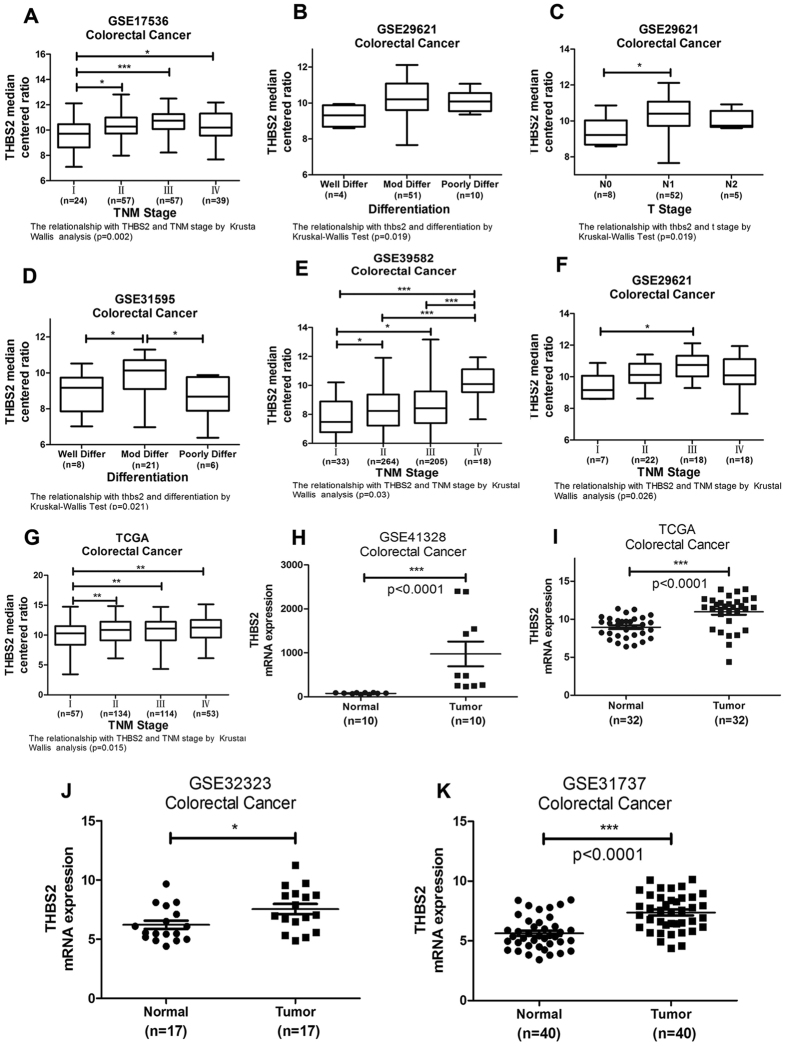
THBS2 was significant correlation with clinicopathology parameters in GEO datasets. (**A**) Krustal-Wallis Test confirmed that the THBS2 was correlation with TNM stage and differentiation. GSE17536, TNM stage, p = 0.002; (**B**) GSE29621, differentiation, p = 0.019; (**C**) GSE29621, T stage, p = 0.019; (**D**) GSE31595, differentiation, p = 0.021; (**E**) GSE39582, TNM stage, p = 0.030; (**F**) GSE29621, TNM stage, p = 0.026 (**G**) TCGA, TNM stage, p = 0.015; (**H**) GSE41328 showed that THBS2 was higher expression in tumor tissue when compared with paired adjacent normal tissue (p < 0.001); (**I**) TCGA showed that THBS2 was higher expression in tumor tissue when compared with paired adjacent normal tissue (p < 0.001); (**J**) GSE32323 showed that THBS2 was higher expression in tumor tissue when compared with paired adjacent normal tissue (p = 0.05); (**K**) GSE31737 showed that THBS2 was higher expression in tumor tissue when compared with paired adjacent normal tissue (p < 0.001).

To demonstrate the portability and repeatability prognostic value of THBS2, we obtained a validation cohort from the GEO and TCGA datasets. Kaplan–Meier survival analysis was performed to evaluate the prognostic value of the Gene signature in twelve Affymetrix datasets retrieved from the GEO database. The log-rank test results confirmed that the THBS2 was closely related to OS in nine datasets and DFS/RFS in thirteen datasets (Fig. 2; (A) GSE16125-OS, n = 32, p = 0.033; (B) GSE17536-OS, n = 177, p < 0.001; (C) GSE17536-RFS, n = 145, p < 0.001; (D) GSE17537-OS, n = 55, p = 0.076; (E) GSE17537-RFS, n = 42, p = 0.204; (F) GSE24549-GPL11028-DFS, n = 83, p = 0.002; (G) GSE24549-GPL5175-DFS, n = 95, p = 0.261; (H) GSE24550-GPL5175-DFS, n = 77, p = 0.018; (I) GSE24550-GPL11028-DFS, n = 77, p = 0.711; (J) GSE28722-OS, n = 125, p = 0.123; (K) GSE28722-MFS, n = 125, p = 0.016; (L) GSE29621-OS, n = 65, p = 0.039; (M) GSE31595-RFS, n = 37, p = 0.388; (N) GSE33113-RFS, n = 89, p = 0.053; (O) GSE38832-OS, n = 122, p = 0.175; (P) GSE38832-DFS, n = 92, p = 0.005; (Q) GSE39582-OS, n = 579, p < 0.001; (R) GSE39582-RFS, n = 519, p < 0.001; (S) GSE56699-OS, n = 61, p = 0.994; (T) GSE56699-DFS, n = 61, p = 0.629; (U) TCGA, n = 361, p = 0.012; (V) TCGA-RFS, n = 311, p = 0.007). Krustal-Wallis Test confirmed that THBS2 was correlated with TNM stage and differentiation (Fig. 3; (A) GSE17536, TNM stage, p = 0.002; (B) GSE29621, differentiation, p = 0.019; (C) GSE29621, T stage, p = 0.019; (D) GSE31595, differentiation, p = 0.021; (E) GSE39582, TNM stage, p = 0.030; (F) GSE29621, TNM stage, p = 0.026; (G) TCGA, TNM stage, p = 0.015). Datasets GSE41328, TCGA, GSE32323 and GSE31737 showed that THBS2 was higher expression in tumor tissue when compared with paired adjacent normal tissue (Fig. 3H–K). Above results suggested that THBS2 was a cancer-related gene in CRC. The correlation between THBS2 expression with clinicopathology parameters was showed in Supplementary Table S6.

### THBS2 levels are associated with poor patient survival in clinical samples

**Figure 4:**
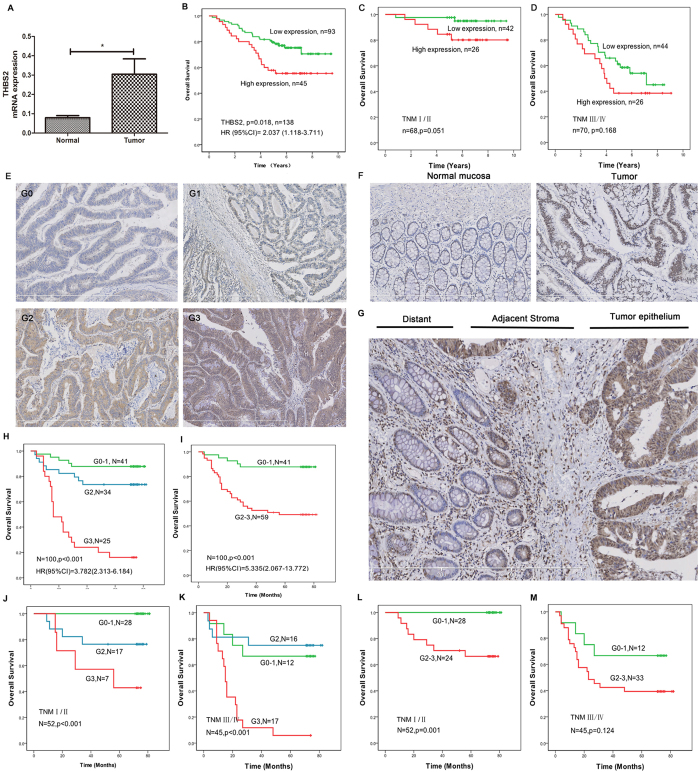
Expression of THBS2 in clinical colorectal tissues. (**A**) THBS2 mRNA expression in colorectal cancer tissues and paired adjacent normal tissues examined by qRT-PCR and normalized to GAPDH. (**B**) THBS2 high-expression predicts a poor prognosis for CRC patients by Kaplan-Meier survival curve. (**C**) Overall survival of THBS2 mRNA expression in TNM stage I/II. (**D**) Overall survival of THBS2 mRNA expression in TNM stage III/IV. (**E**) Representative images of THBS2 IHC of biopsies from human patients with CRC. Staining patterns were categorized into four grades (G0–G3). Scale bars, 600 um. (**F**) THBS2 protein expression in colorectal cancer tissues and paired normal mucosa examined by IHC. (**G**) THBS2 protein expression in distant, adjacent stroma and tumor epithelium. (**H**) Overall survival curves of G0-1, G1 versus G3 groups. (**I**) Overall survival curves of G0–G1 versus G2-3 groups. (**J**) Overall survival curves of G0-1, G1 versus G3 in TNM stage I/II. (**K**) Overall survival curves of G0-1 versus G2-3 in TNM stage III/IV. (**L**) Overall survival curves of G0-1 versus G2-3 in TNM stageI/II. (**M**) Overall survival curves of G0-1 versus G2-3 in TNM stage III/IV.

The clinical samples showed that THBS2 was significant higher expression in tumor tissue when compared with paired adjacent normal tissue (Fig. 4A). Significantly correlations with several clinicopathology parameters were observed in tumor THBS2 expression (Table 1). Mann-Whitney test analysis showed that age-related reduction in THBS2 expression can be found in the patient with age more than 60 (p = 0.048). Gender seems to be an important factor that affect the expression of THBS2, female showed higher expression of THBS2 than male (Median = 0.0498, Median = 0.0283, respectively). Moreover, increased level of THBS2 was significantly associated with advanced infiltrating depth, lymph node metastasis and TNM stage (P = 0.044, P = 0.050, P = 0.033, respectively). Meanwhile, the expression of THBS2 was partly associated with differentiation, although it didn’t reach statistic significance when incorporate all of the differentiation levels into consideration (P = 0.327). There was no statistical difference between THBS2 level with location, tumor size, grade and metastases (all *P* > 0.05). However, there still existed some expression difference in location and distant metastasis. The expression of THBS2 in rectrum or metastasis patients showed a higher level.

At the end of this study, 43 patients died of CRC among 138 patients. The overall ten year survival rate (OS) of patients with high THBS2 was 59.7%, lower than the patients with low expression. As shown Fig. 4B, Kaplan–Meier analysis revealed that the CRC patients with higher level of THBS2 was significantly associated with a poor overall survival (median, 6.54 years) than those with lower level patients (median, 7.77 years, *P* = 0.018, HR (95% CI) = 2.037(1.118–3.711)). Moreover, there was a significant correlation between THBS2 mRNA expression and overall survival in different TNM stage, especially in early TNM stage. Patients with higher THBS2 mRNA expression were associated with the poor overall survival in TNM stage I/II (p = 0.051, Fig. 4C). Although there was no statistical difference between THBS2 mRNA expression with overall survival in TNM stage III/IV, the higher level of THBS2 showed a higher risk(p = 0.168, Fig. 4D). We used the multivariate cox proportional hazards regression model to analyze the independent prognostic value of THBS2 by adjusting location, differentiation, infiltrating depth, lymph node metastasis, distant metastasis and TNM stage. After adjustment, THBS2 still showed a significant prognostic value that higher expression of THBS2 showed a higher risk in overall survival (p = 0.022, HR (95% CI) = 2.093(1.110–3.947)).

We further detect THBS2 protein expression level of 100 CRC tissue samples including 10 paired cases with both normal and tumor samples by immunohistochemistry (IHC). The results were graded from 0 to 3 (G0, G1, G2, G3) depending on average number of THBS2-positive aggregates per field (Fig. 4E). As shown in Fig. 4F, THBS2 was over expressed in tumor when compared with paired normal tissues. The expression level of THBS2 showed a gradually increasing trend from distant tissues, adjacent stroma to tumor epithelium (Fig. 4G). And the THBS2 expression showed a significant positive correlation with age (*P* = 0.024), lymph node metastasis (*P* = 0.008) and distant metastasis (*P* = 0.046). In addition, there was a significant positive correlation between THBS2 expression and the tumor TNM grade (*P* = 0.001), (Table 2). However, no correlations between THBS2 expression and gender, site, tumor size, histological type, general type and differentiation were found. Consistently with the results of mRNA, Overall survival was much worse in patients with high grade THBS2 in tumors than in patients with low or no THBS2 staining (Fig. 4H,I). Combined with TNM stage, the higher THBS2 expression was associated with the worse overall patient survival (Fig. 4J,L) in TNM stage I/II. But there was no statistical significance for overall survival between high and low grade THBS2 staining in TNM stage III/IV, even that just G3 grade THBS2 staining showed worst overall survival (Fig. 4K,M). Taken together, these results indicated that THBS2 was a prognostic factor independent of TNM stage, especially in TNM stage I/II. Multivariate Cox analysis revealed THBS2 as an independent prognostic factor after adjustment with age, sex, differentiation, lymph node metastasis, distant metastasis and TNM stage (P < 0.001, Table 2).

### Meta-analysis confirmed the validity of THBS2

**Figure 5:**
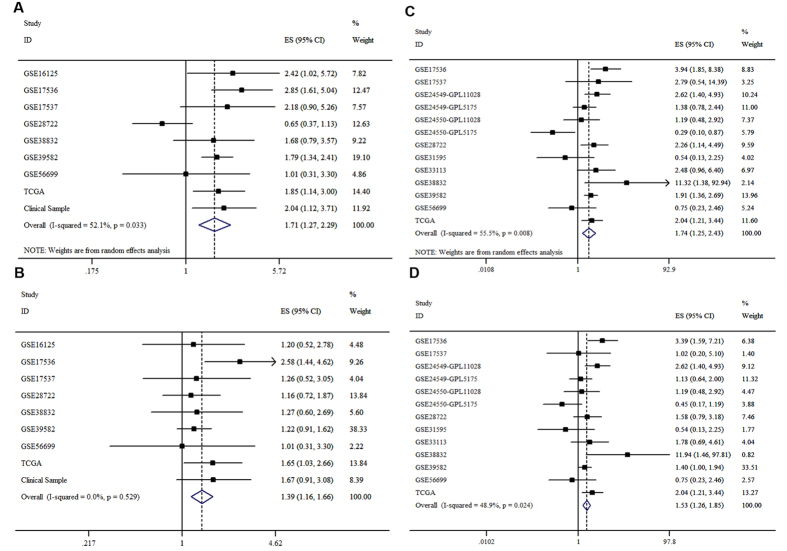
Forest plot of the association between THBS2 and CRC survival. Meta-analysis of THBS2 in seven independent datasets was conducted, and HR, 95% CI of each gene and corresponding p value were calculated and plotted in the forest plot. (**A**) Forest plot of the association between THBS2 filtered by ROC with a random-effect model in datasets containing OS information. (**B**) Forest plot of the association between THBS2 filtered by p50 with a fixed-effect model in datasets containing OS information. (**C**) Forest plot of the association between THBS2 filtered by ROC with a random-effect model in datasets containing DFS information. (**D**) Forest plot of the association between THBS2 filtered by p50 with a random-effect model in datasets containing DFS information.

Meta-analysis was conducted to evaluate the correlation between THBS2 and survival of CRC patients with fixed-effect and random-effect models. We used ROC as an evaluation for THBS2 expression, which showed that the higher THBS2 expression with higher HR in overall survival. The I-squared correlation of THBS2 expression with HR is 52.1%; Heterogeneity chi-squared = 16.70, p = 0.033; Estimate of between-study variance Tau-squared = 0.0956; Test of ES = 1: Correlation coefficient z = 3.57, p < 0.001; HR (95% CI) = 1.709(1.273–2.294) (Fig. 5A). P50 was used as another evaluation, the result showed that the I-squared correlation of THBS2 expression with HR is = 0.0%; Heterogeneity chi-squared = 7.07, p = 0.529; Test of ES = 1: Correlation coefficient z = 3.62, p < 0.001; HR (95% CI) = 1.366(1.162–1.657) (Fig. 5B). Data showed that the higher expression mean high HR in DFS/RFS using ROC as an evaluation for THBS2 expression, and the I-squared correlation of THBS2 expression with HR is 55.5%, Estimate of between-study variance Tau-squared = 0.1737; Test of ES = 1: Correlation coefficient z = 3.30, p = 0.001; Heterogeneity chi-squared = 26.95, p = 0.008; HR (95% CI) = 1.743(1.253–2.425) (Fig. 5C). Data showed that the I-squared correlation of THBS2 expression with HR is 48.9% using p50 as an evaluation; Heterogeneity chi-squared = 23.49, p = 0.024; Test of ES = 1: Correlation coefficient z = 4.35, p < 0.001; HR (95% CI) = 1.526(1.261–1.847) (Fig. 5D). Funnel chart showed in Supplement material Supplementary Figure S2.

### THBS2 promoted proliferation and metastasis in CRC

**Figure 6:**
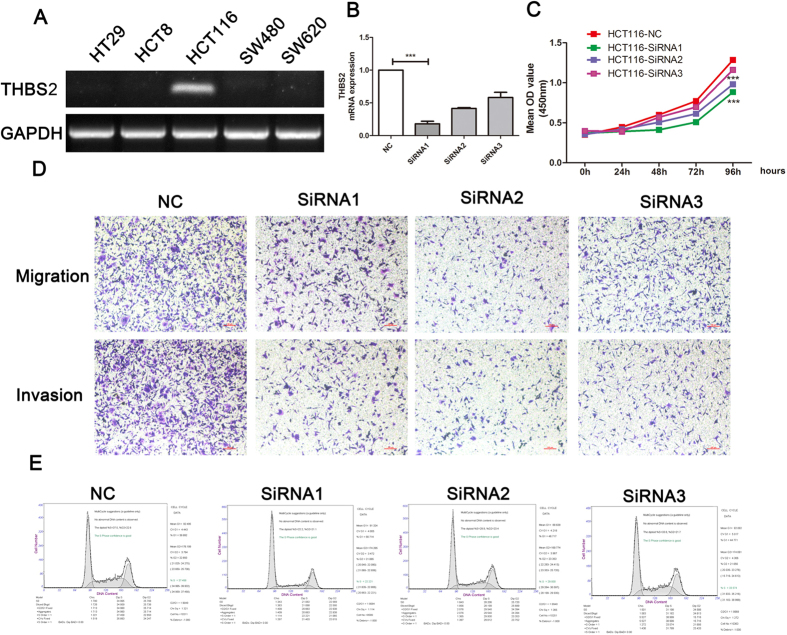
THBS2 promotes proliferation and metastasis in HCT116 cell. (**A**) The expression of THBS2 in colorectal cancer cell lines. (**B**) Detection of THBS2 in HCT116 cell with THBS2 knockdown by qRT-PCR. (**C**) Cell proliferation for HCT116 with THBS2 knockdown. (**D**) Migration and invasion assay for HCT116 with THBS2 knockdown. (**E**) Cell cycle with THBS2 knockdown.

To clarify the biological roles of THBS2 in CRC, we knocked down the expression of THBS2 in HCT116 cell line expressing high level THBS2 (Fig. 6A,B). CCK8 assay showed that THBS2 knockdown inhibited cell proliferation (Fig. 6C). Transwell assay showed that migration and invasion ability were decreased by siRNA for THBS2 (Fig. 6D). Otherwise, THBS2 silencing could also induce G1/S cell cycle arrest (Fig. 6E). Together with these results, THBS2 might promote colorectal cancer growth and metastasis.

## Discussion

Cancer is one of the most common leading causes of death worldwide. Prognostic at an early stage is a useful way that decrease and avoid mortality. Although remarkable progress has been made to investigate the underlying mechanism, the understanding of the complicated carcinogenesis process was enormously hindered by large-scale tumor heterogeneity. Here we proposed that the diagnose-related genes, responsible for cooperativity disorientation, probably contain untapped prognostic resource of colorectal cancer. The current study investigated a comprehensive evaluation method application in profound prognostic value gene in colorectal cancer and attempted to evaluate a new prognostic candidate gene THBS2 underlying significant value. Multiple analysis indentified THBS2 as an independent prognostic factor for survival. High level of THBS2 was associated with poor disease-free and overall survival in a cohort of serous colorectal cancer patients that integrated data combination GEO/TCGA datasets with meta-analysis, suggesting that THBS2 may be a prognostic biomarker of colorectal cancer. The expression of THBS2 was significant correlated with lymphatic invasion and TNM stage in CRC patients.

Base on the GEO dataset GSE17536, we found some specificity or universality genes between recurrence and non-recurrence patients. The significant differently expressed genes included 56 up-regulated and 3 down-regulated genes, indicated the up-regulated genes maybe the dominant factor that affected the progress of cancer recurrence. In order to analyze the biological and statistical significance of DEGS, we constructed a PPI network that included degree distribution and neighborhood score for the 59 genes. Most node distributions were between 0–6, only 4 genes’ degrees were more than 6. The neighborhood score showed that MEP1A, MFAP5, THBS2, VCAN, FBN1 and COMP owned the highest score, which indicated these genes might play important functions.

To further evaluate the biological significance for DEGs, we performed KEGG pathway enrichment analysis for molecular functions significantly enriched Focal adhesion, ECM-receptor interaction and TGF-beta pathways^19^,^20^. Interestingly, several of these genes such as THBS2 and COMP that filtered after strict condition attracted our attention. They were specificity genes that high expression in recurrence patients when compared with non-recurrence patients. They own the same degree distribution and extremely high neighborhood score. Furthermore, they showed significant additive effect on the whole pathways. Above results suggested that THBS2 and COMP were important recurrence-related genes in CRC.

In previous study, Kalmár A. *et al*. reported that THBS2 showed hypomethylation in both colorectal adenoma and CRC-normal adjacent tissue^21^. Lin D. *et al*. found that COMP and THBS2 in plasma of colon cancer patients were higher expression than non-cancer control^22^. Hoon Kim *et al*. indentified that THBS2 could be used for developing high specificity biomarkers sensing cancer invasion and predicting response to neoadjuvant therapy, as well as potential multi-cancer metastasis inhibiting therapeutics targeting the corresponding biological mechanism^23^. THBS2 showed partly correlation with colorectal cancer, COMP reported rarely in colorectal cancer, it was mostly associated with arthritis^24^,^25^. The function of these genes involved in CRC development has not been discovered.

By analyzing the associations between the expression profiles of THBS2 and COMP, clinical outcome of CRC patients and healthy volunteers in GEO datesets, although the expression of COMP correlated with prognosis outcome, it did not present significant difference expression in tumor tissue when compared with paired adjacent normal tissue from two datasets. As a universality gene in tumor and normal tissue, COMP might not be considered as a biomarker associated with colorectal cancer.

We found that THBS2 was significantly up regulated in tumor tissue when compared with paired adjacent normal tissue. The correlation between THBS2 expression with survival was significant, regardless of the data evaluated as a continuous variable or category variable. A clear separation was observed in the survival curves between patients with low- or high-risk signatures. Patients with a low-risk THBS2 signature in their tumor specimens tended to have prolonged overall survival, whereas patients with a high-risk signature tended to have shortened survival. The usefulness of THBS2 could be internally validated in the non-overlapping mostly GEO datasets, indicating good reproducibility of this gene signature in the overall survival of CRC. However, there still existed some datasets show a nonsense result, even one dataset showed the opposite result. The disease-free survival also showed the similar phenotype. Therefore, a systematic integration of gene expression data from multiple sources meta-analyses as a comprehensive evaluation has been used for THBS2. ROC is a normalized method in data category variable, especially in improving data significance and expanding difference. Another analysis used P50 as a filter.

Fortunately, after the comprehensive evaluation method application in discovery gene signature with profound prognostic value in colorectal cancer, THBS2 have been reserved and demonstrated by clinical samples. Our study presents the first line of evidences that THBS2 expression is up-regulated mRNA and increased THBS2 expression is associated with poor overall survival of colorectal cancer. In order to further identify the clinical role of THBS2 in CRC patients, we measured the protein expression of THBS2 in CRC tissues specimens through immunohistochemistry. We found that THBS2 expression was positively correlated with lymph node metastasis, distant metastasis and clinical stage. Our study suggested the high level of THBS2 expression serves as a significant role in CRC progression through promoting tumor growth and accelerating tumor cell metastasis. Differentiation, infiltrating depth, lymph node metastasis, distant metastasis and TNM stage were currently the most important known prognostic factor. Among the host factors, age was a powerful prognostic factor. Elderly patients had poorer survival in cancer, partly because of comorbidities that caused lower response to therapy^26^. The current study also showed an attributable to age. In addition, gender was also an important factor that associated with cancer specificity. Chih-Feng Chian *et al*. reported several genes had been indentify to be gender-dependent markers^27^. Among the disease factors, increasing data suggest that the tumor size have independent significance and could supplement prognostication^28^,^29^. Although there was no statistical difference between THBS2 level with gender, location, tumor size, grade and differentiation, these factors might have potentially diagnose value. We still found some slightly expression difference in part clinicopathology parameters. The expression of THBS2 in rectrum or mod/poor differentiation patients showed a higher level. These findings suggest that THBS2 could play a potentially critical role of in the pathogenesis and progression of colorectal cancer. With the gradual depth studies, THBS2 might become as a potential biomarker for predicting clinical outcome for colorectal cancer patients.

## Methods

### Discovery and Extraction of differentially expressed genes

We used Affymetrix data set publicly available in GEO database^30^ with available clinical information as originally research. GSE17536 data set includes individual gene expression level with overall survival, disease-specific survival and disease-free survival information for 177 patients with CRC disease collected at the Moffitt Cancer (United States), and it was used to define the molecular classification. The recurrence-associated genes were determined by limma package in Bioconductor that based on: (1) fold change > 0.5; (2) false discovery rate based on the moderated t test followed by Benjamini and Hochberg’s multiple-test adjustment <0.05. Directional concordance between recurrence patients and non-recurrence patients from VMC with poor outcome was determined to refine 59-gene recurrence classifier.

### Hierarchical cluster and Neighborhood Scoring

Cluster analysis was performed to examine the extracted genes with significantly different levels of expression between patients with diverse outcomes in recurrence group and non-recurrence group. The expression data of the probes corresponding to protein in the Retrieval of Interacting Genes (STRING)^31^ terms from functional annotation analysis were extracted to establish the network. Neighborhood Scoring was based on the distribution and fold change of different expressed genes in network^32^.

### Pathway analysis and Hypergeometric Distribution

DAVID (http://david.abcc.ncifcrf.gov/) Pathway Analysis was used for functional annotation of the transcripts and identification of upstream regulators. The hypergeometric distribution test was used to examine the correlation between gene with disease that measured by the P-value.

### Public datasets analysis

CRC expression profiling studies including relevant clinical information were identified by searching public datasets. The following key words and their combinations were used: “colon cancer or colorectal cancer and expression profiling by array” to search in GEO datasets (Supplement Figure S1). In addition, dataset from TCGA was also searched to ensure the relevant studies were not missed. Datasets with gene expression profile comparing CRC or colorectal adenoma to paired adjacent normal tissue were obtained from Dataset GSE32323 which contained 17 paired samples. And the comparison between 40 paired colorectal adenoma and adjacent normal tissue samples were performed by dataset GSE31737. The GSE33113 data set includes disease-free survival information for 90 patients with AJCC stage II disease collected at the Academic Medical Center in Amsterdam (the Netherlands). GSE39582 includes expression and clinical data for 566 patients with CRC collected for the Cartesd’Identité des Tumeurs (CIT) program, from the French LigueNationaleContrele Cancer. Other datasets that contained complete clinical information were also be used to generate survival curves of THBS2. Other datasets that contained complete clinical information were also been used to analysis the survival curves of THBS2 (Supplementary Table S7).

### Clinical specimens

This study was approved by Zhejiang University’s School of Medicine and was carried out in accordance with the approved guidelines and regulations. All experimental protocols were approved by Zhejiang University’s School of Medicine. All participants provided written, informed consent for this study and the ethics committee of Zhejiang University’s School of Medicine approved the study. 138 CRC patients were recruited from Sir Run Run Shaw Hospital of Zhejiang University. 110 CRC tissues including 10 paired normal tissues were recruited from affiliated hospital, Zhejiang University School of Medicines and Sir Run Run Shaw Hospital of Zhejiang University, respectively. The histologically normal tissues in the distant margin to the tumor were collected at the time of surgery from patient who undergoing resection of colorectal tumors. Pathologic diagnoses were evaluated by pathologists via biopsy reports and patients with familial adenomatous polyposis, hereditary non-polyposis CRC and inflammatory bowel disease were excluded. All tissue samples were obtained from colorectal adenocarcinoma patients without any adjuvant treatment including radiotherapy or chemotherapy prior to surgery and diagnosis.

### RNA extraction and quantitative RT-PCR

Total RNA from the tissues were extracted using TRIZOL Reagent (Invitrogen). The RNA concentration was determined using UV spectrophotometry. RT-PCR was performed using Thunderbird SYBR Master Mix (Takara, Japan). The PCR was performed on a Real-time PCR Detection System (StepOnePlus, ABI) with the following cycles: 95 °C for 1 min, followed by 40 cycles of 95 °C for 15 s, 60 °C for 15 s, and 72 °C for 45 s to detect the THBS2 and GAPDH gene levels. GAPDH expression was used as an internal control. GAPDH-Sense: ACCACAGTCCATGCCATCAC; GAPDH-Antisense: TCCACCACCCTGTTGCTGTA. THBS2-Sense: TCCTGCTGGCTCTGTGGGTGT; THBS2-Antisense: ATGGTCTTGCGGTTGATGTTGCT. The 2^−ΔCT^ was calculated for every sample and normalized to GAPDH. All RT-PCR results are representative of three independent experiments.

### Immunohistochemistry analysis

Immunohistochemistry (IHC) study was carried out as described previously^33^. The THBS2 antibody was obtained from Sangon biotech (1:200 for IHC).

### Statistical analysis

The statistical package SPSS (version 20.0; IBM New York, NY, USA) was applied. Unpaired Student’s t tests were used for normally distributed data and non-parametric Mann-Whitney U-tests were used for non-normally distributed data to compare central tendencies. For results in CRC tissues, Relapse-free, metastasis-free, or overall survival was compared between high and low THBS2 expression groups using median gene expression value as a bifurcating point. Correlations were analyzed by the Spearman coefficient test. Significance was set at P < 0.05. Stata software was used to be as a comprehensive evaluation that associated public datasets with clinical samples.

### Cell lines and cell culture

The human CRC cell lines HCT116 were purchased from the American Type Culture Collection (Manassas, VA, USA), which were maintained in RPMI 1640 medium; The cell was routinely maintained and supplemented with 10% fetal bovine serum (HyClone, Tauranga, New Zealand) and grown at 37 °C in an atmosphere of 95% air and 5% CO_2_.

### RNA interference

SiRNA targeting humanTHBS2 and one negative control siRNA were designed and synthesized by Genepharma (Shanghai, China). Transfection of each siRNA (50 nM) was carried out using Lipofectamine2000 (Invitrogen) according to the manufacturer’s recommendations.

THBS2-SiRNA1: GUUUGCUUCAGAACGUCCATT

  UGGACGUUCUGAAGCAAACTT

THBS2-SiRNA2: GUUGGCAAUAUCACACGCATT

  UGCGUGUGAUAUUGCCAACTT

THBS2-SiRNA3: CAAUGAACGAGACAAUUGUTT

  ACAAUUGUCUCGUUCAUUGTT

NC: UUCUCCGAACGUGUCACGUTT

  ACGUGACACGUUCGGAGAATT

### Cell viability and cycle assays

Cell viability was assessed by CCK-8 (Boster, Wuhan, China). Cycle level was measured by flow cytometry by staining the cells with propidium iodide (Multi Sciences, Hangzhou, China).

### Cell migration and invasion assay

To evaluate the migration and invasion capacity of HCT116, 24-well plates equipped with cell culture inserts containing 8.0 μm pore size membrane (Costar Corp., Cambridge, MA, USA) were used. For invasion assay, diluted extracellular matrix gel (BD Biosciences, Bedford, MA, USA) coated the inserts preincubated for 30 min. 1 × 10^5^ cells incubated for the migration assay and 2 × 10^5^ cells incubated for the invasion assay. At the end of the experiments, cells on the lower surface of the filters were fixed in 4% paraformaldehyde and stained by 0.1% crystal violet.

## Additional Information

**How to cite this article**: Wang, X. *et al*. THBS2 is a Potential Prognostic Biomarker in Colorectal Cancer. *Sci. Rep.* **6**, 33366; doi: 10.1038/srep33366 (2016).

## Supplementary Material

Supplementary Information

## Figures and Tables

**Table 1:** Mann-Whitney test and cox proportional hazards regression model to assess the correlation between the clinical features with the expression of THBS2 and overall survival.

Characteristics	Number (n = 138)	Median expression of THBS2	*P*_*a*_ value	Overall Survival	
				*P*_*b*_ value	*HR (95% CI)*_*b*_
Age (years)					
≤60	44	0.0766	**0.048**	0.856	1.057(0.582–1.921)
>60	94	0.0291			
Gender					
Male	72	0.0283	0.139	0.234	0.708(0.400–1.251)
Female	66	0.0498			
Location					
Colon	62	0.0314	0.864	**0.032**	0.540(0.307–0.948)
Rectrum	76	0.0453			
Tumor size (cm)					
≤5	69	0.0391	0.920	0.214	1.431(0.813–2.521)
>5	69	0.0362			
Differentiation					
Well	103	0.0137	0.327	0.151	1.274(0.916–1.772)
Mod	14	0.1377			
Poor	20	0.0298			
Unknown	1				
Grade					
N0	117	0.0391	0.869	0.963	1.018(0.477–2.172)
N1	21	0.0362			
Infiltrating Depth					
N0	19	0.0226	**0.044**	**<0.001**	3.334(2.012–5.524)
N1	89	0.0278			
N2	30	0.1011			
Lymph node metastasis					
M0	79	0.0305	**0.05**	**<0.001**	3.333(1.832–6.065)
M1	59	0.0484			
Distant Metastasis					
N0	113	0.0350	0.498	**<0.001**	5.813(3.279–10.306)
N1	25	0.0450			
TNM stage					
I/II	68	0.0278	**0.033**	**<0.001**	8.223(3.686–18.344)
III/IV	70	0.0497			
**THBS2 expression**			***P***_***c***_	***P***_***d***_	***HR***(***95% CI***)_***d***_
Low	93	0.0136	**0.018**	**0.022**	2.093(1.110–3.947)
High	45	0.3000			

(1) *P*_*a*_ value for the correlation between clinicopathological variables with THBS2 expression. (2) *P*_b_ value for the correlation between clinicopathological variables with overall survival. (3) *P*_*c*_ value for the correlation between THBS2 expression with survival determined with the log rank test. (4) *P*_*d*_ value was measured by multivariate analyses of overall survival (Cox proportional hazards regression model) after adjustment age, sex, location, differentiation, infiltrating depth, lymph node metastasis, distant metastasis and TNM stage.

**Table 2:** Relationship between THBS2 protein expression and clinicopathologic characteristic of CRC patients.

Characteristic	Number*	Cases of death (%)	*P*_*a*_ value	*HR (95% CI*)	Expression of THBS2			*P*_*b*_ value
					G0-1	G2	G3	
Age (yr)			0.256	1.513(0.741–3.091)				**0.014**
≤60	40	11(27.5%)			19	18	3	
>60	60	24(40.0%)			22	16	22	
Gender			0.317	1.403(0.723–2.722)				0.976
Male	57	18(31.6%)			23	20	14	
Female	43	17(39.5%)			18	14	11	
Site			0.249	0.646(0.307–1.358)				0.497
Colon	59	23(40.0%)			25	16	18	
Rectum	39	10(25.6%)			15	18	6	
Size (cm)			0.997	1.001(0.498–2.013)				0.806
≤5	53	18(34.0%)			23	16	14	
>5	39	14(35.9%)			14	16	9	
Histological type			0.417	1.393(0.6250–3.102)				0.907
Tubular adenocarcinoma	77	24(31.2%)			33	25	19	
Others	19	8(42.1%)			7	8	4	
General type			0.979	0.990(0.489–2.006)				0.424
Ulcerated	56	19(33.9%)			23	16	17	
Others	37	13(35.1)			15	16	6	
Differentiation			**0.001**	3.158(1.610–6.195)				0.389
Well/moderate	75	20(26.7%)			31	28	16	
Poor/undifferentiated	25	15(60%)			10	6	9	
Lymph node metastasis			**0.001**	3.402(1.646–7.030)				**0.008**
Negative	53	11(20.8%)			27	17	9	
Positive	42	22(52.4%)			11	16	15	
Distant metastasis			**0.002**	3.520(1.559–7.947)				**0.046**
Negative	80	22(27.5%)			36	27	17	
Positive	11	8(72.7%)			2	4	5	
TNM stage			**<0.001**	4.649(2.084–10.374)				**0.001**
I/II	52	8(15.4%)			28	17	7	
III/IV	45	24(53.3%)			12	16	17	
Expression of THBS2			**<0.001**	3.782(2.313–6.184)	**Multivariate COX**			
G0-1	41	5(12.2%)			***P***_***c***_ **value**		***HR (95% CI)***	
G2	34	9(26.5)			**<0.001**		3.164(1.698–5.927)	
G3	25	21(84%)						

(1) *P*_*a*_ values for the cox proportional hazards regression model analysis the correlation between clinicopathologic characteristic with overall survival. (2) *P*_*b*_ value for the chi-squared test analysis the correlation between clinicopathologic characteristic. (3) *P*_*c*_ value for the multivariate COX proportional hazard model analysis the diagnose value of THBS2 after adjustment age, sex, differentiation, lymph node metastasis, distant metastasis and TNM stage.

^*^Some data were missed.
