# Supplementary material for: THBS2 is a Potential Prognostic Biomarker in Colorectal Cancer
Source: Sci Rep. 2016 Sep 16;6:33366. doi: 10.1038/srep33366 (PMC5025892; doi:10.1038/srep33366)
Supplement: Supplementary Information [file srep33366-s1.doc]

**THBS2 is a Potential Prognostic Biomarker in Colorectal Cancer**

Xue Wang ^[1]^, Lei Zhang ^[1]^, Hui Li^[2]^,WenJie Sun^[2]^, Honghe Zhang^[2,3]*^,MaodeLai ^[1, 2,3]*^

Affiliation:

[1] Department of Pharmacology, China Pharmaceutical University, Nanjing, 210009, China

[2]Department of Pathology, School of Medicine, Zhejiang University, Hangzhou 310058, China

[3]Key Laboratory of Disease Proteomics of Zhejiang Province, Hangzhou 310058, China

*Corresponding author: Dr.HZhang, Department of Pathology, School of Medicine, Zhejiang University, Hangzhou 310058, China.Tel: +86-571-88208199; Fax: +86-571-88208198, E-mail: [honghezhang@zju.edu.cn](mailto:honghezhang@zju.edu.cn); And Prof.M Lai, Department of Pharmacology, China Pharmaceutical University and Department of Pathology, School of Medicine, Zhejiang University, Hangzhou 310058, China. Tel: +86-571-88208199; Fax: +86-571-88208198, E-mail: [lmp@zju.edu.cn](mailto:lmp@zju.edu.cn)

**Supplementary material**

**
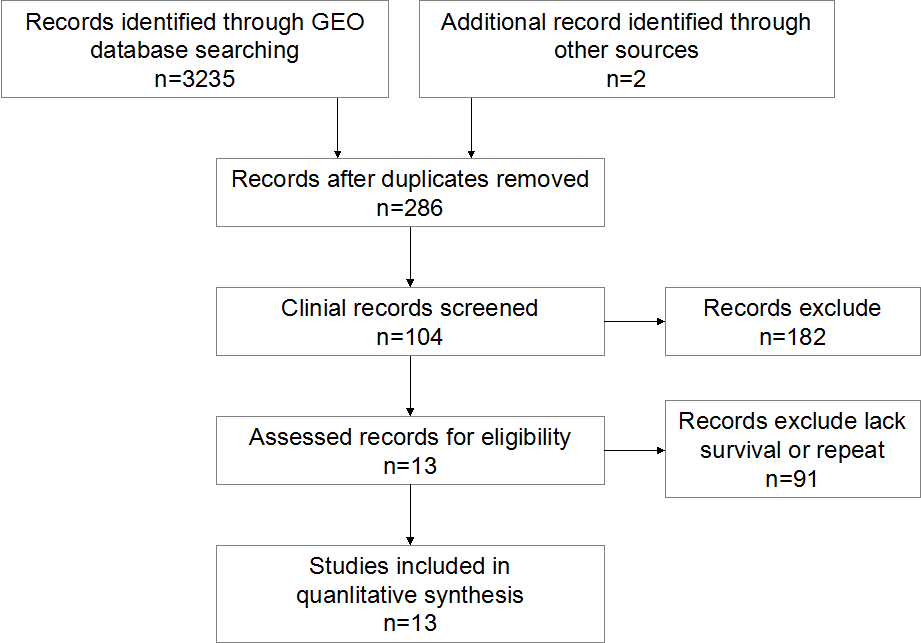
**

**Supplementary Figure S1. The searching process of datasets that associated with THBS2.**

**
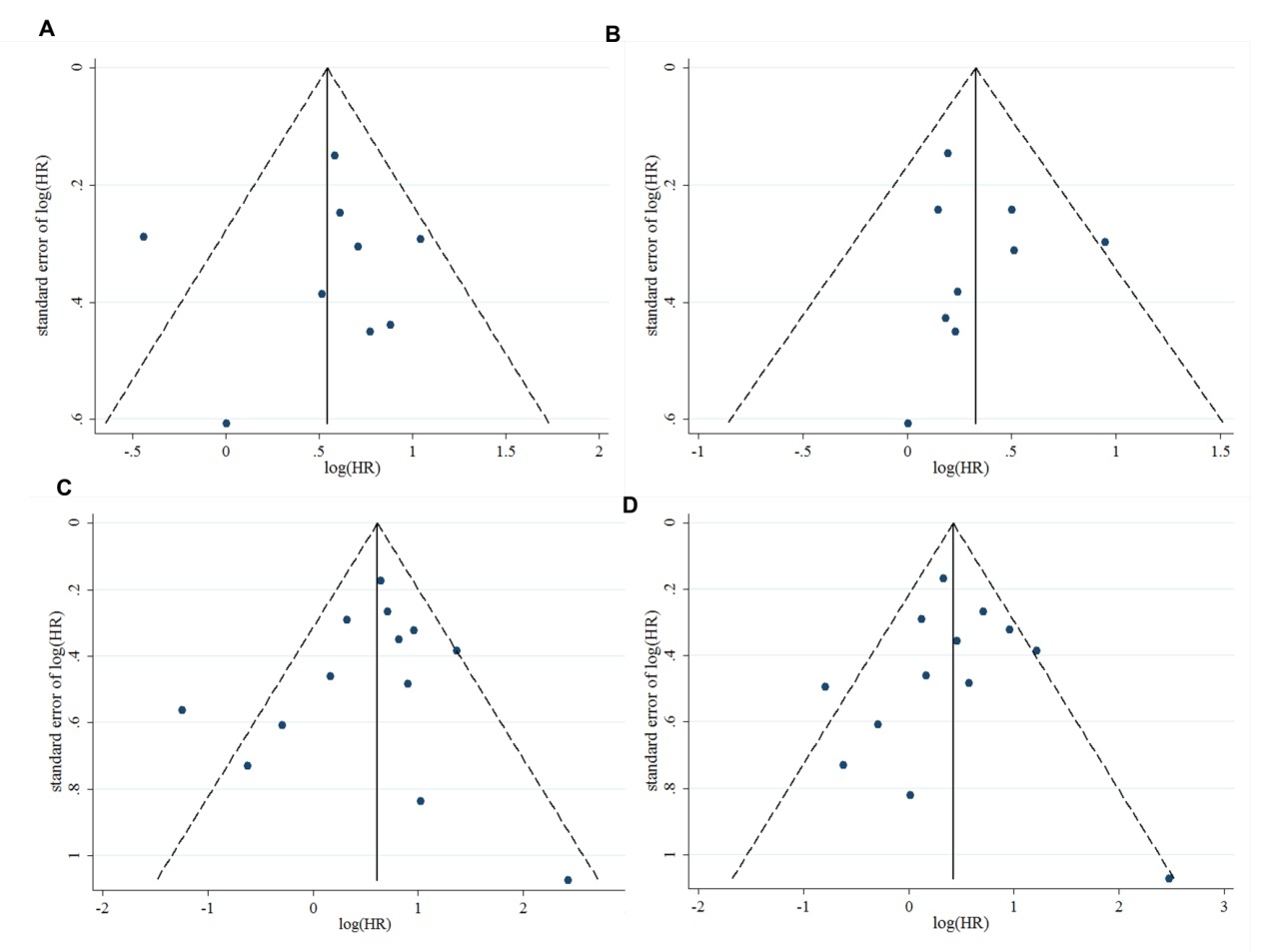
**

**Supplementary Figure S2. Funnel charts (A) Overall survival by ROC (B) Overall survival by p50 (C) Disease-free survival by ROC (D) Disease-free survival by p50**

| Gene | Log FC | AveExpr | T | P.Value | Adj.P.Val |
| --- | --- | --- | --- | --- | --- |
| ADAMTS5 | 0.50760306 | 6.641571771 | 6.136241906 | 7.59E-09 | 8.79E-05 |
| CYP1B1 | 0.977016748 | 6.870662941 | 5.547861762 | 1.33E-07 | 0.001023937 |
| STC1 | 0.567457754 | 7.710920189 | 5.4406545 | 2.19E-07 | 0.001269841 |
| NOX4 | 0.52230475 | 5.75371619 | 5.241147442 | 5.50E-07 | 0.002511368 |
| BGN | 0.646079056 | 8.926209269 | 5.204329568 | 6.51E-07 | 0.002511368 |
| SFRP2 | 1.369443749 | 9.561945772 | 5.126681736 | 9.24E-07 | 0.003000502 |
| COL11A1 | 1.103054388 | 8.223033975 | 5.030724378 | 1.42E-06 | 0.003000502 |
| THBS2 | 0.923217174 | 10.35173737 | 5.002733113 | 1.61E-06 | 0.003000502 |
| SERPINE1 | 0.507713509 | 7.829586685 | 4.975210707 | 1.81E-06 | 0.003000502 |
| PRRX1 | 0.76798779 | 7.231159179 | 4.807248223 | 3.77E-06 | 0.004010824 |
| OLFML2B | 0.634006686 | 8.481556648 | 4.760296 | 4.62E-06 | 0.004650166 |
| PCOLCE2 | 0.549810695 | 5.732179834 | 4.720788698 | 5.47E-06 | 0.004767327 |
| GFPT2 | 0.502247336 | 7.348981193 | 4.707974821 | 5.77E-06 | 0.00477604 |
| RAI14 | 0.52050824 | 9.085837503 | 4.639796817 | 7.71E-06 | 0.005577562 |
| MFAP5 | 0.977005151 | 7.422884793 | 4.610354916 | 8.72E-06 | 0.005941245 |
| KANK4 | 0.534232775 | 7.001979828 | 4.583927663 | 9.74E-06 | 0.006421568 |
| INHBA | 0.59181653 | 8.525884701 | 4.555707811 | 1.10E-05 | 0.006421568 |
| FN1 | 0.570592392 | 10.1223637 | 4.537578415 | 1.18E-05 | 0.0065165 |
| HTRA1 | 0.601673594 | 9.983133186 | 4.515935791 | 1.29E-05 | 0.006676011 |
| GAS1 | 0.653367663 | 7.406736034 | 4.515094781 | 1.30E-05 | 0.006676011 |
| CCL11 | -0.601751164 | 8.186745041 | -4.495102178 | 1.41E-05 | 0.006692531 |
| VCAN | 0.719335099 | 10.29675583 | 4.472314452 | 1.55E-05 | 0.006892019 |
| COL10A1 | 1.168408113 | 9.30081071 | 4.460156651 | 1.63E-05 | 0.007108182 |
| ZFPM2 | 0.626471296 | 6.195225655 | 4.452511069 | 1.68E-05 | 0.007198745 |
| PXDN | 0.548031923 | 9.272557272 | 4.360106537 | 2.44E-05 | 0.009362793 |
| POSTN | 0.52542057 | 8.504881016 | 4.358675503 | 2.46E-05 | 0.009362793 |
| COMP | 0.910684156 | 8.076035793 | 4.356366711 | 2.48E-05 | 0.009362793 |
| LOXL1 | 0.608867809 | 8.35017151 | 4.251513309 | 3.78E-05 | 0.0122078 |
| VGLL3 | 0.57927582 | 6.536399231 | 4.21672099 | 4.33E-05 | 0.013126115 |
| COL5A2 | 0.620933732 | 10.77309443 | 4.199115303 | 4.65E-05 | 0.013559653 |
| FRMD6 | 0.661871224 | 8.714886328 | 4.096699059 | 6.93E-05 | 0.016937098 |
| FBN1 | 0.618585218 | 8.48238769 | 4.040012431 | 8.62E-05 | 0.019344093 |
| C5AR1 | 0.637247164 | 8.289413786 | 4.012034071 | 9.59E-05 | 0.020142106 |
| PPAPDC1A | 0.621359927 | 7.908995828 | 4.001208567 | 1.00E-04 | 0.020531985 |
| CTGF | 0.587221669 | 10.27395027 | 3.978050732 | 0.00010915 | 0.02124397 |
| JUP/KRT17 | 0.715392916 | 7.782366724 | 3.917817182 | 0.000136976 | 0.024207105 |
| LOX | 0.587951309 | 7.252947864 | 3.877113353 | 0.000159471 | 0.024614498 |
| IGFL2 | 0.516633922 | 8.185343283 | 3.855121033 | 0.000173044 | 0.025366294 |
| COL5A1 | 0.550379039 | 9.883257356 | 3.839659331 | 0.000183237 | 0.026147886 |
| APOE | 0.513901357 | 9.534963529 | 3.809147158 | 0.000205047 | 0.027856237 |
| SLC2A3 | 0.564722599 | 8.5353684 | 3.78115727 | 0.000227201 | 0.030417375 |
| IBSP | 0.553407457 | 6.928102521 | 3.774012213 | 0.00023321 | 0.030626228 |
| CDH11 | 0.510304801 | 8.64829964 | 3.742148365 | 0.000261889 | 0.032404621 |
| SPOCK1 | 0.705172056 | 8.133351255 | 3.713582903 | 0.000290411 | 0.034493357 |
| SRPX | 0.544656973 | 8.091506262 | 3.698016058 | 0.000307167 | 0.03575023 |
| MATN3 | 0.837898307 | 4.823315034 | 3.680501127 | 0.000327113 | 0.036561747 |
| EPYC | 1.037485286 | 5.952541407 | 3.666672484 | 0.000343719 | 0.037374988 |
| MGP | 0.541768856 | 7.647052107 | 3.646151969 | 0.000369836 | 0.038584553 |
| ADAM12 | 0.750260676 | 7.825202607 | 3.635575655 | 0.000384018 | 0.038724512 |
| MEP1A | -0.981090056 | 9.497594938 | -3.633350001 | 0.000387067 | 0.038724512 |
| IGJ | -0.984437033 | 11.13910652 | -3.629156508 | 0.000392874 | 0.038724512 |
| AEBP1 | 0.55742816 | 9.934466269 | 3.620717685 | 0.000404811 | 0.039229405 |
| ITGBL1 | 0.526466243 | 6.145679251 | 3.604815242 | 0.000428241 | 0.040649508 |
| TMEM45A | 0.64717164 | 8.456697234 | 3.583672552 | 0.000461376 | 0.042404483 |
| TIMP3 | 0.507400615 | 9.74108771 | 3.559580038 | 0.000502077 | 0.045072095 |
| OLR1 | 0.791885843 | 7.019174214 | 3.5583995 | 0.000504156 | 0.045083967 |
| GEM | 0.528972744 | 9.420413476 | 3.551960279 | 0.000515638 | 0.045718764 |
| FNDC1 | 0.742561907 | 9.118702207 | 3.530463229 | 0.000555779 | 0.046825498 |
| RAB31 | 0.543003097 | 9.869706409 | 3.519916799 | 0.000576531 | 0.047519713 |

**Supplementary Table S1. Recurrence associated genes from DEGs**

| Gene | NS score | LogFC | Degree | adj.P.Val |
| --- | --- | --- | --- | --- |
| MEP1A | 0.78 | -0.981090056 | 2 | 0.038724512 |
| MFAP5 | 0.64 | 0.977005151 | 3 | 0.005941245 |
| COMP | 0.6 | 0.910684156 | 3 | 0.009362793 |
| FBN1 | 0.48 | 0.618585218 | 6 | 0.019344093 |
| VCAN | 0.46 | 0.719335099 | 4 | 0.006892019 |
| THBS2 | 0.46 | 0.923217174 | 3 | 0.003000502 |
| FN1 | 0.4 | 0.570592392 | 9 | 0.0065165 |
| SPOCK1 | 0.35 | 0.705172056 | 3 | 0.034493357 |
| FRMD6 | 0.33 | 0.661871224 | 2 | 0.016937098 |
| BGN | 0.32 | 0.646079056 | 4 | 0.002511368 |
| LOXL1 | 0.3 | 0.608867809 | 2 | 0.0122078 |
| HTRA1 | 0.3 | 0.601673594 | 3 | 0.006676011 |
| LOX | 0.29 | 0.587951309 | 2 | 0.024614498 |
| CTGF | 0.29 | 0.587221669 | 2 | 0.02124397 |
| COL5A1 | 0.28 | 0.550379039 | 2 | 0.026147886 |
| APOE | 0.26 | 0.513901357 | 2 | 0.027856237 |
| GEM | 0.26 | 0.528972744 | 2 | 0.045718764 |
| RAI14 | 0.26 | 0.52050824 | 2 | 0.005577562 |
| TIMP3 | 0.25 | 0.507400615 | 4 | 0.045072095 |
| SERPINE1 | 0.25 | 0.507713509 | 3 | 0.003000502 |

**Supplementary Table S2 DEGs degree distribution and Neighborhood Scoring**

| Term | Count | P-Value | Benjamini |
| --- | --- | --- | --- |
| ECM-receptor interaction | 7 | 2.70E-07 | 6.50E-06 |
| Focal adhesion | 7 | 4.40E-05 | 5.30E-04 |
| TGF-beta signaling pathway | 3 | 3.70E-02 | 2.60E-01 |

**Supplementary Table S3 Pathway enrichment**

|  | Gene | Disease | filter | P value | Pubmed ID |
| --- | --- | --- | --- | --- | --- |
| Gene | COMP | CRC | overlap | 0.995601 | 24224610 23567896 |
| number | 1037 | 182043 | 2 |
| ratio | 4.15E-05 | 7.28% | 8.00E-08 |
| Gene | THBS2 | CRC | overlap | 0.002995 | 26482433 24224610 22200551 21047417 |
| number | 81 | 182043 | 4 |
| ratio | 3.24E-06 | 7.28% | 1.60E-07 |

**Supplementary Table S4 Hypergeometric Distribution**

| Correlated gene | | Pearson’correlation | | Correlated gene | | Pearson’correlation | | |
| --- | --- | --- | --- | --- | --- | --- | --- | --- |
| BGN | | 0.928973627 | | NRG1 | | -0.457144179 | | |
| CTHRC1 | | 0.926912563 | | CTA | | -0.460222468 | | |
| COL11A1 | | 0.923774238 | | NIPAL1 | | -0.466533147 | | |
| SPARC | | 0.91839788 | | BCL2L14 | | -0.475303885 | | |
| ADAM12 | | 0.913901509 | | LOC100506379 | | -0.476818215 | | |
| COL5A2 | | 0.91253875 | | IL17A | | -0.491203455 | | |
| INHBA | | 0.912449895 | | BMP5 | | -0.499189944 | | |
| RAB31 | | 0.911257196 | | DNASE1L3 | | -0.540727559 | | |
| COL10A1 | | 0.908652802 | | COL4A6 | | -0.564142744 | | |
| AEBP1 | | 0.907014351 | | NSG1 | | -0.607027057 | | |
|  | Overall Survival | | | | | | | |
| The average of  correlated gene | P_a_ value | | HR(95%CI)_a_ | | P_b_ value | | HR(95%CI)_b_ | |
|  | <0.001 | | 2.746(1.593-4.734) | | <0.001 | | 3.052(1.729-5.387) | |
|  | Disease-free Survival | | | | | | | |
|  | P_a_ value | | HR(95%CI)_a_ | | P_b_ value | | HR(95%CI)_b_ | |
|  | <0.001 | | 3.448(1.746-8.811) | | 0.001 | | 3.361(1.686-6.700) | |
|  | Overall Survival | | | | | | | |
| The average of  correlated gene and THBS2 | P_a_ value | | HR(95%CI)_a_ | | P_b_ value | | HR(95%CI)_b_ | |
|  | <0.001 | | 2.890(1.676-4.981) | | <0.001 | | 3.166(1.797-5.577) | |
|  | Disease-free Survival | | | | | | | |
|  | P_a_ value | | HR(95%CI)_a_ | | P_b_ value | | | HR(95%CI)^b^ |
|  | <0.001 | | 3.448(1.746-8.811) | | 0.001 | | | 3.361(1.686-6.700) |

**Supplementary Table S5 Pearson correlation and cox proportional hazards regression model analysis.** (1)*Pa* value for the correlation between THBS2 expression with survival. (4) *Pb* value was measured by multivariate analyses of overall survival (Cox proportional hazards regression model) after adjustment age, sex and TNM stage.

| Characteristics | Number   (n=177) | Median expression  of THBS2 | HR (95%CI) | P value | |
| --- | --- | --- | --- | --- | --- |
| Age(years) |  |  |  | |  |
| ≤60 | 59 | 10.5825 | 0.746 (0.445-1.312) | | 0.217 |
| >60 | 118 | 10.3304 |  | |  |
| Gender |  |  |  | |  |
| Male | 96 | 10.3729 | 0.843 (0.491-1.446) | | 0.764 |
| Female | 81 | 10.4343 |  | |  |
| Recurrence |  |  |  | |  |
| N0 | 109 | 10.1118 | 42.869 (10.159-180.896) | | <0.001 |
| N1 | 36 | 11.2229 |  | |  |
| Grade |  |  |  | |  |
| Well differentiated | 16 | 9.9354 | 2.133 (1.241-3.665) | | 0.589 |
| Mod differentiated | 134 | 10.4023 |  | |  |
| Poorly differentiated | 27 | 10.4343 |  | |  |
| TNM stage |  |  |  | |  |
| Ⅰ | 24 | 9.7017 | 3.623 (2.495-5.262) | | 0.002 |
| Ⅱ | 57 | 10.2750 |  | |  |
| Ⅲ | 57 | 10.7448 |  | |  |
| Ⅳ | 39 | 10.1999 |  | |  |

**Supplementary Table S6** Mann-Whitney test to assess the correlation between THBS2 levels with clinical features in GSE17536.

| GEO datasets | Study design | Country | Platforms | Counts | Analysis |
| --- | --- | --- | --- | --- | --- |
| GSE31737 | prospectively adenomas/normal mucosa | USA | GPL5175 | 80 | expression |
| GSE32323 | prospectively adenomas/normal mucosa | Japan | GPL570 | 34 | expression |
| GSE41328 | prospectively adenomas/normal mucosa | USA | GPL570 | 10 | expression |
| GSE16125 | Retrospective | Italy | GPL5175 | 36 | OS |
| GSE17536 | Retrospective | USA | GPL570 | 177 | OS/DFS |
| GSE17537 | Retrospective | USA | GPL570 | 55 | OS/DFS |
| GSE24549 | Retrospective | Norway | GPL5175 | 95 | DFS |
|  |  |  | GPL11028 | 83 | DFS |
| GSE24550 | Retrospective/normal mucosa | Norway | GPL5175 | 90 | DFS |
|  |  |  | GPL11028 | 77 | DFS |
| GSE28722 | Retrospective | USA | GPL13425 | 125 | DFS |
| GSE29621 | Retrospective | USA | GPL570 | 65 | Corresponding to GSE17536 |
| GSE31595 | Retrospective | Denmark | GPL570 | 37 | DFS |
| GSE33113 | Retrospective | Netherlands | GPL570 | 96 | DFS |
| GSE38832 | Retrospective | USA | GPL570 | 122 | OS/DFS |
| GSE39852 | Retrospective | France | GPL570 | 585 | OS/DFS |
| GSE56699 | Retrospective | Italy | GPL14951 | 72 | OS |
| TCGA | Retrospective/normal mucosa |  |  | 635 | OS/DFS |
| Clinical | Retrospective/normal mucosa | China |  | 138 | OS |
|  | Retrospective | China |  | 100 | OS |
|  | prospectively adenomas/normal mucosa | China |  | 20 | expression |

**Supplementary Table S7 The datasets associated with colorectal cancer.**
